# Persistent Object Search and Surveillance Control With Safety Certificates for Drone Networks Based on Control Barrier Functions

**DOI:** 10.3389/frobt.2021.740460

**Published:** 2021-10-25

**Authors:** Hayato Dan, Takeshi Hatanaka, Junya Yamauchi, Takumi Shimizu, Masayuki Fujita

**Affiliations:** ^1^ School of Engineering, Tokyo Institute of Technology, Tokyo, Japan; ^2^ Guraduate School of Information Physics and Computing, The University of Tokyo, Tokyo, Japan

**Keywords:** search and surveillance, drone networks, safe control, persistency, control barrier functions, distributed control, coverage control

## Abstract

In this paper, we address a persistent object search and surveillance mission for drone networks equipped with onboard cameras, and present a safe control strategy based on control barrier functions The mission for the object search and surveillance in this paper is defined with two subtasks, persistent search and object surveillance, which should be flexibly switched depending on the situation. Besides, to ensure actual persistency of the mission, we incorporate two additional specifications, safety (collision avoidance) and energy persistency (battery charging), into the mission. To rigorously describe the subtask of persistent search, we present a novel notion of *γ*-level persistent search and the performance certificate function as a candidate of a time-varying Control Barrier Function. We then design a constraint-based controller by combining the performance certificate function with other CBFs that individually reflect other specifications. In order to manage conflicts among the specifications, the present controller prioritizes individual specifications in the order of safety, energy persistency, and persistent search/object surveillance. The present controller is finally demonstrated through simulation and experiments on a testbed.

## 1 Introduction

**FIGURE 1:**
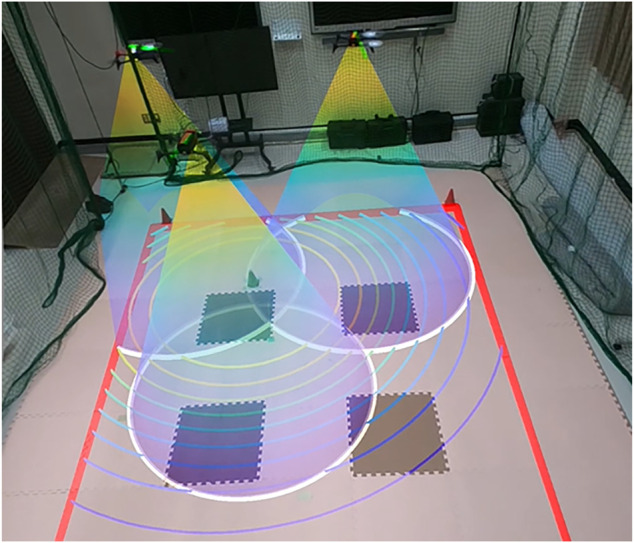
A scene of coverage control with drone networks.

Environmental monitoring is one of the key applications of networked multi-robot systems, wherein each robot is expected to deploy over the mission space. To this end, the most promising control technology is coverage control that provides distributed control strategies for enhancing efficiency of information acquisition on the environment (Cortés et al., 2005; Martínez et al., 2007; Renzaglia et al., 2012). The recent technological advances in drone technology make it viable to implement coverage control on drone networks, and many successful results have been reported in the literature (Schwager et al., 2011; Bentz et al., 2018; Funada et al., 2019). These publications consider the scene such that drones with onboard cameras looking down the ground to be monitored move around over the ground as illustrated in Figure 1.

Specifications for environmental monitoring vary depending on the application scenarios. In this paper, we address a scene where drones are required to surveil a target object on the environment whose location is initially unknown for the drones. In this scenario, the drones need to first search the object, and then to switch the task to surveillance of the object once it is found. In the phase of searching, the drones are expected to take exploratory actions to patrol the mission space while avoiding too much overlaps of fields of view among drones. Avoiding the overlaps is handled by coverage control but most of the coverage control algorithms lead robots to a stationary configuration rather than persistently taking patrolling motion. Consequently, some subregion may remain uncovered and, accordingly, the drones may fail to find the object especially when the number of drones is not many enough to fully cover the environment as in the scene of Figure 1. To address the issue, the authors presented persistent coverage control schemes in (Hübel et al., 2008; Sugimoto et al., 2015; Kapoutsis et al., 2019), where a notion of information reliability is introduced and so-called density function is dynamically updated according to the reliability. It is then exemplified that the gradient ascent algorithm with the update of the density function generates persistently patrolling motion over the mission space. A similar concept is also presented in (Wang and Wang, 2017), wherein the concept is termed *awareness*. However, these methodologies do not provide any guarantee on the coverage performance. Meanwhile, Franco et al. (2015) and Palacios-Gasós et al. (2016) address the performance guarantee for the persistent coverage, but a prescribed performance level is not always ensured therein in the presence of the performance decay in time. Kapoutsis et al. (2019) present a persistent coverage scheme not requiring exact models of the environment and robot’s coverage capabilities.

In order to ensure persistency of the mission in practice, it is not enough just to make drones take persistent motion and we have to meet a variety of constraints. For example, we need to certify safety during the mission. Specifically, collision avoidance among drones must be a key in ensuring persistency since drones no longer continue the mission if they collide with each other just once. Moreover, drones are normally driven by batteries with limited storage, and battery exhaustion prevents drones from continuing the mission. We thus need to take account of energy persistency, namely we need to control drones so that they return to charging stations before their batteries are exhausted. These issues have been individually addressed e.g. in (Hussein et al., 2007; Zhu and Martínez, 2013; Bentz et al., 2018; Wang et al., 2020), but a more general framework to flexibly integrate a variety of specifications is needed. Meanwhile, a great deal of recent publications have been devoted to Control Barrier Function (CBF) in order to certify the constraint fulfillment, e.g., to ensure safe operation of multi-robot systems (Ames et al., 2017; Notomista et al., 2018). The CBF has also been employed in coverage control, e.g., in (Egerstedt et al., 2018; Funada et al., 2019). Egerstedt et al. (2018) certifies collision avoidance and maintenance of the energy level in the coverage mission based on the inherent flexibility of CBFs that allows one to integrate various specifications. Funada et al. (2019) manages overlaps of fields of view for drone networks using the CBFs. The paper most closely related to the present paper is Santos et al. (2019), wherein the authors investigate coverage control with a time-varying density function similarly to the persistent coverage control. However, the paper does not give any explicit guarantee of the coverage performance.

In this paper, we present a novel persistent object search and surveillance control with safety certificates for drone networks based on CBFs. We first introduce a new concept of *γ*-level persistent search as a performance metric for the searching mission in the form of a constraint function. We then formulate constraint functions that describe the control goal for the object surveillance and specifications for safety (collision avoidance) and energy persistency (battery charging). We then formulate inequality constraints to be met by the control input, following the manner of CBFs. A constraint-based controller is then presented, including all of the above inequality constraints. The controller with all of the constraints however may result in issues on infeasibility in online optimization required by the controller. We thus present prioritization among the constraints, where we place priority in the order of safety, energy persistency, and persistent search/object surveillance. Based on the designed priority, we present a novel constraint-based controller that ensures feasibility, where the inequality constraints for persistent search and object surveillance are appropriately switched depending on whether the object is detected or not. The controller is moreover shown to be implemented in a partially distributed manner. We then run simulation of the constraint-based control only with the performance certificate for the persistent search. It is revealed there that the present constraint-based controller maintains the *γ*-level persistent search during the simulation, while the gradient-based controller in (Sugimoto et al., 2015) occasionally fails to meet the level. Finally, we implement the present control algorithm including not only the constrained-based controller but also an object detection algorithm and takeoff from/landing to the charging stations on a testbed with three drones.

The contributions of this paper are summarized as follows: 1) a novel constraint-based controller is presented so that a prescribed performance level is maintained, differently from the gradient-based persistent coverage algorithm (Hübel et al., 2008; Sugimoto et al., 2015), constraint-based coverage algorithms (Santos et al., 2019), and other related algorithms (Franco et al., 2015; Palacios-Gasós et al., 2016; Wang and Wang, 2017), 2) a novel object search/surveillance problem is formulated, wherein not only the persistent coverage, safety certificates and energy persistency in (Egerstedt et al., 2018; Santos et al., 2019) but also task switches between search and surveillance are integrated, and 3) the algorithm is demonstrated through experiments, where we put the vision data and associated image processing in the loop while other related publications purely examine only robot motion (Schwager et al., 2011; Sugimoto et al., 2015; Egerstedt et al., 2018; Funada et al., 2019; Santos et al., 2019).

A part of the contents in this paper is presented in the conference version (Dan et al., 2020). The incremental contributions relative to (Dan et al., 2020) are: 4) we implement the present partially distributed control architecture on Robot Operating System (ROS), while the experimental setup in (Dan et al., 2020) took a centralized control architecture, 5) owing to the contribution 4), we increase the number of drones from two to three in the experiment, and 6) we newly add simulation to precisely check if the performance is guaranteed in the absence of uncertain factors in real experiments.

## 2 Preliminary: Control Barrier Function

In this section, we present the notion of control barrier functions that play a central role in this paper. Let us consider a control affine system formulated as

$$\dot{p}=f\left(p\right)+g\left(p\right)u,$$ (1)

where $p\in R^{N},u\in U\subseteq R^{M}$ , and vector fields *f*, *g* are assumed to be Lipschiz continuous. Suppose now that there exists a unique solution *p*(*t*) on [*t*_0_, *t*_1_] to (1). A set S is then said to be *forward invariant* with respect to system (1) if for every $p\left(t_{0}\right)\in S$ , the inclusion p(t)∈S holds for all *t* ∈ [*t*_0_, *t*_1_] (Ames et al., 2017).

Define the Control Barrier Function (CBF) as below.

Definition 1 *Let* $h:D\subset R^{N}\to R$ *be a continuously differentiable function and the set* C *is defined as* $C:=\left\{p\in R^{N}|h\left(p\right)\geq 0\right\}$ *. Then,* *h* *is said to be a CBF for system* (1) *if there exists a locally Lipschitz extended class* K *function* *α* *such that*

$$\underset{u\in U}{\sup}\left[L_{f}h\left(p\right)+L_{g}h\left(p\right)u+\alpha \left(h\left(p\right)\right)\right]\geq 0,$$ (2)

for all *x* in the set C , where *L*_*f*_ *h*(*10*) and *L*_*g*_ *h*(*10*) represent Lie derivative of *h* in the vector fields *f* and *g*, respectively.

It is shown that if *h* is a CBF, then the set C is forward invariant (Ames et al., 2017). If the set C consists of the states that ensure safety, 2) means that there always exists input signal *u* such that the state *p* is enforced to be inside of C , namely safety is always ensured as long as the function *h* that characterizes C is a CBF.

We next present an extension of CBF to the case where the set C is time varying. Consider the following set defined by a continuously differentiable function $h:R^{N}\times R_{\geq t_{0}}\to R$ ,

$$C\left(t\right):=\left\{p\in R^{N}|h\left(p,t\right)\geq 0\right\}.$$ (3)

It is shown that the forward invariance of the set C(t) can be ensured with so-called time-varying CBF defined as follows (Lindemann and Dimarogonas, 2019; Notomista and Egerstedt, 2021).

Definition 2 *Given a dynamical system (1) and a set* C(t) *defined in* Eq. 3 *, the function* *h* *is time-varying CBF defined on* $D\times R_{\geq t_{0}}$ *with* $C\left(t\right)\subseteq D\subset R^{N}$ *, if there exists a locally Lipschitz extended class* K *function* *α* *such that* ∀p∈D *and* ∀*t* ∈ [*t*_0_, *t*_1_]*,*

$$\underset{u\in U}{\sup}\left[\frac{\partial h}{\partial t}+L_{f}h\left(p,t\right)+L_{g}h\left(p,t\right)u+\alpha \left(h\left(p,t\right)\right)\right]\geq 0$$

holds.

## 3 Problem Setting

**FIGURE 2:**
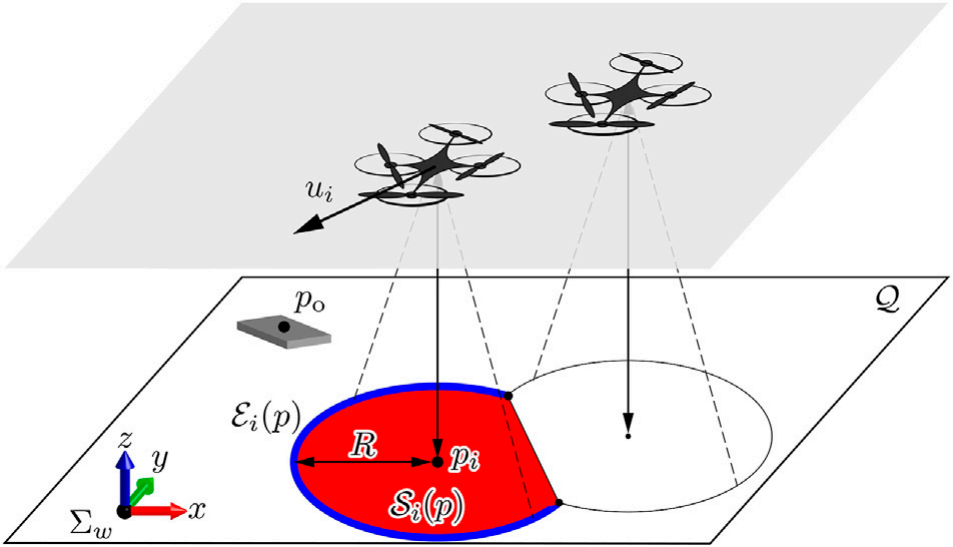
Illustration of the problem setting with the field Q , the world frame Σ_*w*_, drones on the plane (gray plane), drone *i*’s sensing region $S_{i}$ (red region), its inner edge $E_{i}$ (blue curve), and target object.

Let us consider a 3-D space including *n* drones to be controlled and a ground modelled by a 2-D plane as illustrated in Figure 2. Without loss of generality, we arrange the world frame Σ_*w*_ so that its origin is on the ground, and its (*x*, *y*)-plane is parallel to the ground. The subset of the (*x*, *y*)-coordinates on the ground to be monitored is called *field*, and denoted by a compact set $Q\subset R^{2}$ . It is assumed that a target object to be surveilled by the drones may be on the field, and its 2-D position is denoted by $p_{\text{o}}={\left[x_{\text{o}}\;y_{\text{o}}\right]}^{T}\in Q$ . We assume no prior knowledge about not only the position *p*_o_ but also whether the object exists or not. We then define the persistent object search and surveillance mission by the following two subtasks:

• P*ersistent search*: Drones patrol the entire field persistently to search the object.
• *Object surveillance*: Drones keep monitoring the object once the object is found through the persistent search.

These subtasks should be appropriately switched depending on whether the object is detected or not.

Let us denote the set of identifiers of *n* drones by $I=\left\{1,\ldots ,n\right\}$ . The *x*, *y*, and *z* coordinates of drone *i* in Σ_*w*_ are denoted by *x*_*i*_, *y*_*i*_, and *z*_*i*_, respectively. In this paper, each drone is assumed to be locally controlled so that the altitude *z*_*i*_ is constant and common among all drones i∈I . We thus mainly focus on the 2-D motion of $p_{i}={\left[x_{i}\;y_{i}\right]}^{T}\in Q$ . Each drone i∈I is assumed to follow the kinematic model:

$${\dot{p}}_{i}=u_{i},\;u_{i}\in U\subseteq R^{2},$$ (4)

where *u*_*i*_ is the velocity input to be designed. Throughout this paper, we assume that *p*_*i*_ is available for control of drone *i*. Remark that the constant and common altitudes are assumed in order to highlight the main issue to be addressed in this paper. It is actually possible to handle full 3-D motion of the drones, e.g., by taking the formulation of (Funada et al., 2019) at the cost of the computational simplicity.

We next present an external sensor and network models for the drones. Every drone is assumed to be equipped with a single onboard camera that captures the ground. We suppose that the optical axis of each camera is perpendicular to the ground, and that the field of view of camera *i* is modeled by a circle

$$B_{i}\left(p_{i}\right)=\left\{q\in Q|\|q-p_{i}\|\leq R\right\}$$

for a sensing radius *R* > 0. Let us now introduce the Voronoi partition of the field Q (Cortés et al., 2005), which means the collection of the following sets for all i∈I :

$$V_{i}\left(p\right)=\left\{q\in Q|\|q-p_{i}\|\leq \|q-p_{j}\|,\;\forall j\in I\backslash \left\{i\right\}\right\}.$$

Using the above sets, we define the feasible sensing area $S_{i}\left(p\right)$ by so-called *r*-limited Voronoi cell (Martínez et al., 2007) defined by

$$S_{i}\left(p\right):=B_{i}\left(p_{i}\right)\cap V_{i}\left(p_{i}\right),$$

where *p* is the collection of *p*_1_, *p*_2_, … , *p*_*n*_. For convenience of the subsequent discussions, we also define the following set called inner edge of the set $S_{i}\left(p\right)$ (Sugimoto et al., 2015).

$$E_{i}\left(p\right):=\left\{q\in S_{i}|\|q-p_{i}\|=R\right\}.$$

We also assume an inter-drone network such that drone *i* and *j* can exchange messages if their distance ‖*p*_*i*_ − *p*_*j*_‖ is smaller than or equal to 2*R*. It is then well-known that the set $S_{i}\left(p\right)$ is computable in a distributed fashion (Cortés et al., 2005). We also assume that each drone can detect the object when the object is inside of the sensing area $B_{i}\left(p\right)$ , and define a binary variable

$$\Delta_{i}:=\left\{\begin{array}{cc} 1\; & p_{\text{o}}\in B_{i}\left(p\right)\\ 0\; & \text{otherwise}. \end{array}\right.$$

When *Δ*_*i*_ = 1 holds, drone *i* can compute the position of the object *p*_o_ by the detection result and the geometric relation. In real applications, drones need to install an algorithm for detecting the object in the sensing area. See Section 6 for more details on how to detect the object.

In this paper, we implicitly assume that the collection of the fields of view $B_{i}\left(p_{i}\right)$ for all i∈I is not wide enough to fully cover the field Q . The goal of persistent search is then to let the drones persistently patrol the field Q , while preventing any subregion in Q from being uncovered. To address the issue, the authors’ antecessors (Hübel et al., 2008; Sugimoto et al., 2015) presented a gradient ascent algorithm-based controller for the following objective function to be maximized:

$$J\left(p,t\right):=-\overset{n}{\underset{i=1}{\sum }}\int_{S_{i}}\|q-p_{i}{\|}^{2}\phi \left(q,t\right)dq+b\int_{Q\backslash \cup_{i=1}^{n}S_{i}}\phi \left(q,t\right)dq\;\left(b\leq -R^{2}\right),$$ (5)

**FIGURE 3:**
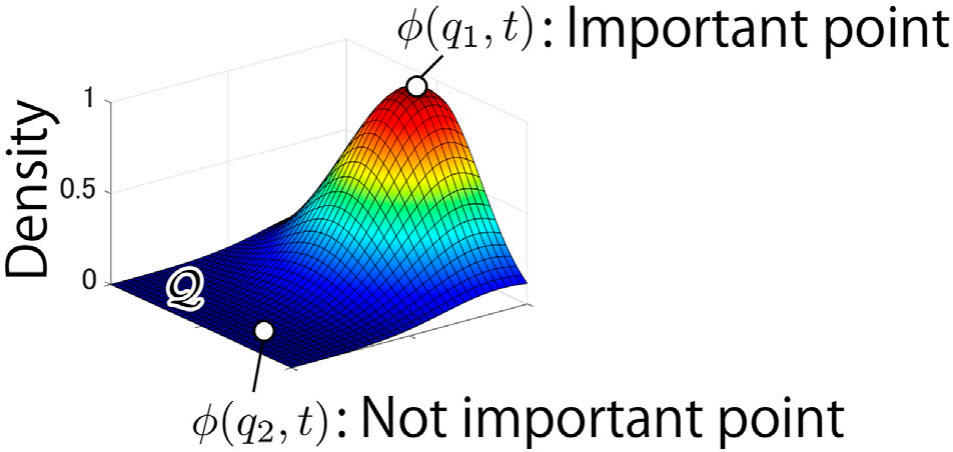
Example of distribution of density function *ϕ*(*q*, *t*). The point *q*_1_ is more important point, which need to be monitored than the point *q*_2_.

The function $\phi :Q\times R_{\geq t_{0}}\to \left[0,1\right]$ , called density function, enables one to mark the important points in the field, as illustrated in Figure 3. The papers (Hübel et al., 2008; Sugimoto et al., 2015) presented a novel update rule of the function *ϕ* formulated by

$$\frac{d\phi \left(q,t\right)}{dt}=\left\{\begin{array}{cc} -\underline{\delta }\phi \left(q,t\right),\; & \text{if}\;q\in \cup_{i=1}^{n}S_{i}\\ \bar{\delta }\left(1-\phi \left(q,t\right)\right),\; & \text{otherwise}. \end{array}\right.\;\left(\bar{\delta },\underline{\delta }>0\right).$$ (6)

Eq. 6 means that importance of point *q* monitored by at least one drone decays while that of point *q* such that $q\notin B_{i}\left(p_{i}\right)\;\forall i\in I$ increases. In view of the nature of the gradient-based coverage, it tends to deliver robots to positions with high density, drones are expected to repeatedly visit all of uncovered regions, which is close to the objective of the persistent search mission in this paper. The control algorithm with Eq. 6 is actually demonstrated through experiments in (Sugimoto et al., 2015). However, the gradient-based controller does not ensure any guarantee on the performance quantified by *J* (*p*, *t*).

In order to certify the search performance, we formally define the objective of the persistent search as below.

Definition 3 *Let a function* $h_{J}:Q^{n}\times R_{\geq t_{0}}\to R$ *be*

$$h_{J}\left(p,t\right):=J\left(p,t\right)-\gamma ,$$

*where* *γ* *is a negative real constant. The drones are then said to achieve* *γ* *-level persistent search, if*

$$h_{J}\left(p\left(t\right),t\right)\geq 0$$ (7)

holds for all *t* ≥ *t*_0_ with a given initial time *t*_0_.

Remark that a similar concept is also investigated in (Franco et al., 2015; Palacios-Gasós et al., 2016). It is an extension of the concept to the time-varying objective function.

Let us next consider the object surveillance that should be performed only when Δ_*i*_ takes the value of 1. Define the function $h_{i,sur}:Q\to R$ as:

$$h_{i,sur}\left(p_{i}\right)=-\|p_{i}-p_{\text{o}}{\|}^{2}+d_{\text{sur}}^{2}.$$

Assuming *R* > *d*_sur_ > 0, the object must be inside of the field of view $B_{i}$ if

$$h_{i,sur}\left(p_{i}\right)\geq 0$$ (8)

holds. It is also fully expected that Eq. 8 holds at the time when Δ_*i*_ switches from 0 to 1. The goal of the object surveillance is thus to keep meeting (8) during the period with Δ_*i*_ = 1.

Besides the above subtasks, we need to meet the following specifications in order to ensure persistency in real operations.

• *Safety*: Drones avoid collisions with each other.
• *Energy persistency*: Drones return to their charging stations before their batteries run out.

If either of the above two would not be satisfied, drones would no longer continue the search and surveillance mission. In this sense, we should place a higher priority on these specifications than 7) and (8). Remark that the subsequent formulations follow the manner of (Egerstedt et al., 2018; Santos et al., 2019).

In order to formulate the specification for safety, let us first define the function:

$$h_{i,avd}\left(p\right)=\|p_{i}-p_{i,near}{\|}^{2}-d_{\text{avd}}^{2},$$

where *p*_*i*,near_ denotes the position of the drone nearest to drone *i* within the radius 2*R*, and *d*_avd_ is selected so that *d*_avd_ > 0. Then, drone *i* keeps the distance from all other drones greater than *d*_avd_ if

$$h_{i,avd}\left(p\right)\geq 0.$$ (9)

holds. Accordingly, collisions are avoided as long as *d*_avd_ is selected to be large enough and 9) is satisfied.

We finally formulate the condition for energy persistency. To this end, the state of charge for drone *i*, denoted by *E*_*i*_, is assumed to obey

$${\dot{E}}_{i}=-K_{\text{chg}}\;\left(K_{\text{chg}}>0\right).$$

We then assume that there is a minimum energy level *E*_min_, that is, *E*_*i*_ ≥ *E*_min_ must hold during the mission. Also, charging stations are assumed to be located on the ground, where the center of the station assigned to drone *i* is denoted by ${\hat{p}}_{i}$ . For simplicity, we leave the landing and takeoff motion out of consideration, and assume that the battery is recharged as long as $\|p_{i}-{\hat{p}}_{i}\|\leq d_{\text{chg}}$ . Let us now define the function

$$h_{i,chg}\left(p_{i},E_{i}\right)=E_{i}-E_{\min}-\frac{K_{\text{chg}}}{k_{\text{chg}}}\left(\|p_{i}-{\hat{p}}_{i}\|-d_{\text{chg}}\right)\;\left(k_{\text{chg}}>0\right).$$

Note that the positive constant *k*_chg_ should be selected so that $\left(K_{\text{chg}}/k_{\text{chg}}\right)\left(\|p_{i}-{\hat{p}}_{i}\|-d_{\text{chg}}\right)$ is greater than the battery needed for returning to the station from the position *p*_*i*_. Then, if the condition

$$h_{i,chg}\left(p_{i},E_{i}\right)\geq 0,$$ (10)

is always satisfied, the state of charge for drone *i* is never exhausted before arriving at the station.

In summary, two subtasks, persistent search and object surveillance, and two specifications, safety and energy persistency, are formulated in the form of the constraint functions (7)–(10), respectively. The control goal for the persistent object search and surveillance mission is to design the control inputs that satisfy the inequalities (7)–(10).

## 4 Constraint-Based Controller

In this section, we present a constraint-based controller to meet (7)–(10) that are possibly conflicting with each other. To this end, we first focus on Eq. 7 for the *γ*-level persistent search in Definition 3.

Now, the time derivative of the function *h*_*J*_ along with the trajectories of system 4) is given as

$${\dot{h}}_{J}=\frac{\partial J\left(p,t\right)}{\partial t}+\overset{n}{\underset{i=1}{\sum }}{\left(\frac{\partial J}{\partial p_{i}}\right)}^{T}u_{i}.$$

The first term in the right hand side of the equation is rewritten as below according to (Diaz-Mercado et al., 2017) and (6).

$$\begin{array}{ll} \frac{\partial J\left(p,t\right)}{\partial t} & =-\overset{n}{\underset{i=1}{\sum }}\int_{S_{i}}\|q-p_{i}{\|}^{2}\frac{\partial \phi \left(q,t\right)}{\partial t}dq+b\int_{Q\backslash \cup_{i=1}^{n}S_{i}}\frac{\partial \phi \left(q,t\right)}{\partial t}dq\\ & =\overset{n}{\underset{i=1}{\sum }}\underline{\delta }\int_{S_{i}}\|q-p_{i}{\|}^{2}\phi \left(q,t\right)dq+b\int_{Q\backslash \cup_{i=1}^{n}S_{i}}\bar{\delta }\left(1-\phi \left(q,t\right)\right)dq. \end{array}$$ (11)

The second term can be expressed as

$$\begin{array}{c} b\int_{Q\backslash \cup_{i=1}^{n}S_{i}}\bar{\delta }\left(1-\phi \left(q,t\right)\right)dq=\overset{n}{\underset{i=1}{\sum }}b\{\frac{1}{n}\int_{Q}\bar{\delta }\left(1-\phi \left(q,t\right)\right)dq-\int_{S_{i}}\bar{\delta }\left(1-\phi \left(q,t\right)\right)dq\}. \end{array}$$ (12)

In the same way as (12), *h*_*J*_ is also rewritten as

$$h_{J}=\overset{n}{\underset{i=1}{\sum }}\{-\frac{\gamma }{n}-\int_{S_{i}}\|q-p_{i}{\|}^{2}\phi \left(q,t\right)dq+\frac{b}{n}\int_{Q}\phi \left(q,t\right)dq-b\int_{S_{i}}\phi \left(q,t\right)dq\}.$$ (13)

Combining (11)–(13), we find that

$${\dot{h}}_{J}+kh_{J}=\frac{\partial J\left(p,t\right)}{\partial t}+\overset{n}{\underset{i=1}{\sum }}{\left(\frac{\partial J}{\partial p_{i}}\right)}^{T}u_{i}+kh_{J}=\overset{n}{\underset{i=1}{\sum }}\left\{{\left(\frac{\partial J\left(p,t\right)}{\partial p_{i}}\right)}^{T}u_{i}+\xi_{i}\left(p,t\right)\right\}\;\;\left(k>0\right),$$

where

$$\begin{array}{ll} \xi_{i}\left(p,t\right) & :=\left(\underline{\delta }-k\right)\int_{S_{i}}\|q-p_{i}{\|}^{2}\phi \left(q,t\right)dq\\ & \;+b\{\frac{1}{n}\int_{Q}\left(\bar{\delta }+\left(k-\bar{\delta }\right)\phi \left(q,t\right)\right)dq-\int_{S_{i}}\left(\bar{\delta }+\left(k-\bar{\delta }\right)\phi \left(q,t\right)\right)dq\}-\frac{k}{n}\gamma . \end{array}$$

Assume that there exists a controller for each agent: $u_{i}=K_{i}\left(p,t\right):Q^{n}\times R_{\geq t_{0}}\to U$ that is locally Lipschitz in $p\in Q^{n}$ , continuous in *t* ∈ [*t*_0_, *t*_1_], and satisfies

$$K_{i}\left(p,t\right)\in K_{i}\left(p,t\right):=\left\{u_{i}\in U|{\left(\frac{\partial J\left(p,t\right)}{\partial p_{i}}\right)}^{T}u_{i}+\xi_{i}\left(p,t\right)\geq 0\right\},$$

$\forall p\in Q^{n}$ and ∀*t* ∈ [*t*_0_, *t*_1_]. This means that the function *h*_*J*_ is a time-varying CBF defined on $Q^{n}\times R_{\geq t_{0}}$ with extended class K function *β*_0_(*s*) = *ks*. Lemma 1 in (Notomista and Egerstedt, 2021) then ensures that the controller guarantees forward invariance of the set

$$C_{0}\left(t\right):=\left\{p\in Q^{n}|h_{J}\left(p,t\right)\geq 0\right\}.$$

Then, the definition of the forward invariance means *γ*-level persistent search for any initial condition inside of the set $C_{0}\left(0\right)$ . In the case of $U=R^{2}$ , $K_{i}\left(p,t\right)=\emptyset$ happens only if $\frac{\partial J\left(p,t\right)}{\partial p_{i}}=0$ . The gradient is now equivalent to the control law in (Sugimoto et al., 2015), wherein $\frac{\partial J\left(p,t\right)}{\partial p_{i}}=0$ means that the robot stops at a point. Through extensive simulations and experiments, we have never observed such a scene and it is fully expected that $K_{i}\left(p,t\right)\neq \emptyset$ in practice, which is demonstrated through simulation in Section 5. Remark also that the above discussions require that the initial state is selected in the set $C_{0}\left(0\right)$ , and do not ensure recovery of the level from an initial condition outside of $C_{0}\left(0\right)$ , namely *h*_*J*_ (*p*, *t*) ≥ 0 for some *t* ≥ *t*_0_ from an initial condition with *h*_*J*_ (*p* (*t*_0_), *t*_0_) < 0. In the case of time-invariant CBFs, the recovery is rigorously proved in (Ames et al., 2017). The result is not trivially extended to the time-varying CBFs. It is however exemplified in Dan et al. (2020) that the recovery is achieved even for the time-varying case in practice.

Let us next consider the satisfaction of Eqs 8–10. It is known that *h*_*i*,sur_, *h*_*i*,avd_ and *h*_*i*,chg_ are all CBFs (Egerstedt et al., 2018; Notomista et al., 2018; Notomista and Egerstedt, 2021). According to Definition 1, we thus formulate the inequality constraints for ensuring (8)–(10) as:

$$\begin{array}{ll} & {\left(\frac{\partial h_{i,sur}}{\partial p_{i}}\right)}^{T}u_{i}+\beta_{1}\left(h_{i,sur}\left(p_{i}\right)\right)=-2{\left(p_{i}-p_{\text{o}}\right)}^{T}u_{i}+\beta_{1}\left(h_{i,sur}\left(p_{i}\right)\right)\geq 0. \end{array}$$ (14)

$$\begin{array}{ll} & {\left(\frac{\partial h_{i,avd}}{\partial p_{i}}\right)}^{T}u_{i}+\beta_{2}\left(h_{i,avd}\left(p_{i}\right)\right)=2{\left(p_{i}-p_{i,\text{near}}\right)}^{T}u_{i}+\beta_{2}\left(h_{i,avd}\left(p_{i}\right)\right)\geq 0, \end{array}$$ (15)

$$\begin{array}{ll} & {\left(\frac{\partial h_{i,chg}}{\partial p_{i}}\right)}^{T}u_{i}-K_{\text{chg}}+\beta_{3}\left(h_{i,chg}\left(p_{i},E_{i}\right)\right)\\ & \;=-{\left(\frac{K_{\text{chg}}\left(p_{i}-{\hat{p}}_{i}\right)}{k_{\text{chg}}\|p_{i}-{\hat{p}}_{i}\|}\right)}^{T}u_{i}-K_{\text{chg}}+\beta_{3}\left(h_{i,chg}\left(p_{i},E_{i}\right)\right)\geq 0, \end{array}$$ (16)

with locally Lipschitz extended class K functions *β*_1_, *β*_2_, *β*_3_ respectively. By definition of CBFs, if we take the controller *u*_*i*_ = *K*_*i*_(*p*, *t*) such that

$$K_{i}\left(p,t\right)\in K_{i}\left(p,t\right):=\{u_{i}\in U^{n}|{\left(\frac{\partial J\left(p,t\right)}{\partial p_{i}}\right)}^{T}u_{i}^{*}+\xi_{i}\left(p,t\right)\geq 0,\left(14\right),\left(15\right),\left(16\right)\},$$

all of Eqs 7–10 are satisfied. However, due to the conflicts among the specifications, the controller set $K_{i}\left(p,t\right)$ can be empty.

To address the above issue, we prioritize the specifications, which can be realized by relaxing some of the constraints. It is now immediate to see that 7) and 8) are never met in practice if the safety constraint 9) or energy constraint (10) is violated. Accordingly to the insight, we propose the following controller *u*_*i*_ = *K*_*i*_(*p*, *t*):

$$\begin{array}{ll} K_{i}\left(p,t\right) & =\underset{\left(u_{i}^{*},\lambda_{i},\mu_{i},\nu_{i}\right)\in U\times R\times R\times R}{\arg\min}\|u_{i}^{*}{\|}^{2}+|\lambda_{i}{|}^{2}+|\mu_{i}{|}^{2}+|\nu_{i}{|}^{2} \end{array}$$ (17a)

$$\begin{array}{ll} s.t.\; & \left(1-\Delta_{i}\right)\left\{{\left(\frac{\partial J\left(p,t\right)}{\partial p_{i}}\right)}^{T}u_{i}^{*}+\xi_{i}\left(p,t\right)\right\}\\ & \;+\Delta_{i}\left\{-2{\left(p_{i}-p_{\text{o}}\right)}^{T}u_{i}^{*}+\beta_{1}\left(h_{i,sur}\left(p_{i}\right)\right)\right\}\geq \epsilon_{\lambda }\lambda_{i}, \end{array}$$ (17b)

$$\begin{array}{ll} & 2{\left(p_{i}-p_{i,\text{near}}\right)}^{T}u_{i}^{*}+\beta_{2}\left(h_{i,avd}\left(p_{i}\right)\right)\geq \epsilon_{\mu }\mu_{i}, \end{array}$$ (17c)

$$\begin{array}{ll} & -{\left(\frac{K_{\text{chg}}\left(p_{i}-{\hat{p}}_{i}\right)}{k_{\text{chg}}\|p_{i}-{\hat{p}}_{i}\|}\right)}^{T}u_{i}^{*}-K_{\text{chg}}+\beta_{3}\left(h_{i,chg}\left(p_{i},E_{i}\right)\right)\geq \epsilon_{\nu }\nu_{i}, \end{array}$$ (17d)

where the weights *ϵ*_*λ*_, *ϵ*_*μ*_, and *ϵ*_*ν*_ are non-negative scalars. The slack variables *λ*_*i*_, *μ*_*i*_, *ν*_*i*_ allow the violations of the associated constraints, and the corresponding weights adjust the penalty on the individual constraint violations. When one of the weights takes a value smaller than other weights, then the controller tries to satisfy the corresponding constraint more strictly than others. When the weight is equal to zero, then the controller treats the constraint as a hard constraint. In this paper, we arrange the weights so that *ϵ*_*λ*_ ≫*ϵ*_*μ*_, *ϵ*_*ν*_ in order to prioritize safety and energy persistency over the control goals of the subtasks. If the weights *ϵ*_*λ*_, *ϵ*_*ν*_, *ϵ*_*μ*_ are all positive or only one of *ϵ*_*μ*_ and *ϵ*_*ν*_ is equal to zero, then the optimization problem in (17) is ensured to be feasible as long as (9) and (10) are satisfied at the initial time *t*_0_.

We finally show that the present controller is implementable in a (partially) distributed manner. The gradient *∂J*(*p*, *t*)/*∂p*_*i*_ in Eq. 17b is known to be rewritten as follows (Cortés et al., 2005):

$$\frac{\partial J\left(p,t\right)}{\partial p_{i}}=2mass\left(S_{i}\left(p\right)\right)\left(cent\left(S_{i}\left(p\right)\right)-p_{i}\right)-\left(R^{2}+b\right)\int_{E_{i}}\frac{q-p_{i}}{\left\|q-p_{i}\right\|}\phi \left(q,t\right)dq,$$

where

$$\begin{array}{ll} mass\left(S_{i}\left(p\right)\right) & :=\int_{S_{i}}\phi \left(q,t\right)dq,\\ cent\left(S_{i}\left(p\right)\right) & :=\frac{1}{mass\left(S_{i}\left(p\right)\right)}\int_{S_{i}}q\phi \left(q,t\right)dq. \end{array}$$

**FIGURE 4:**
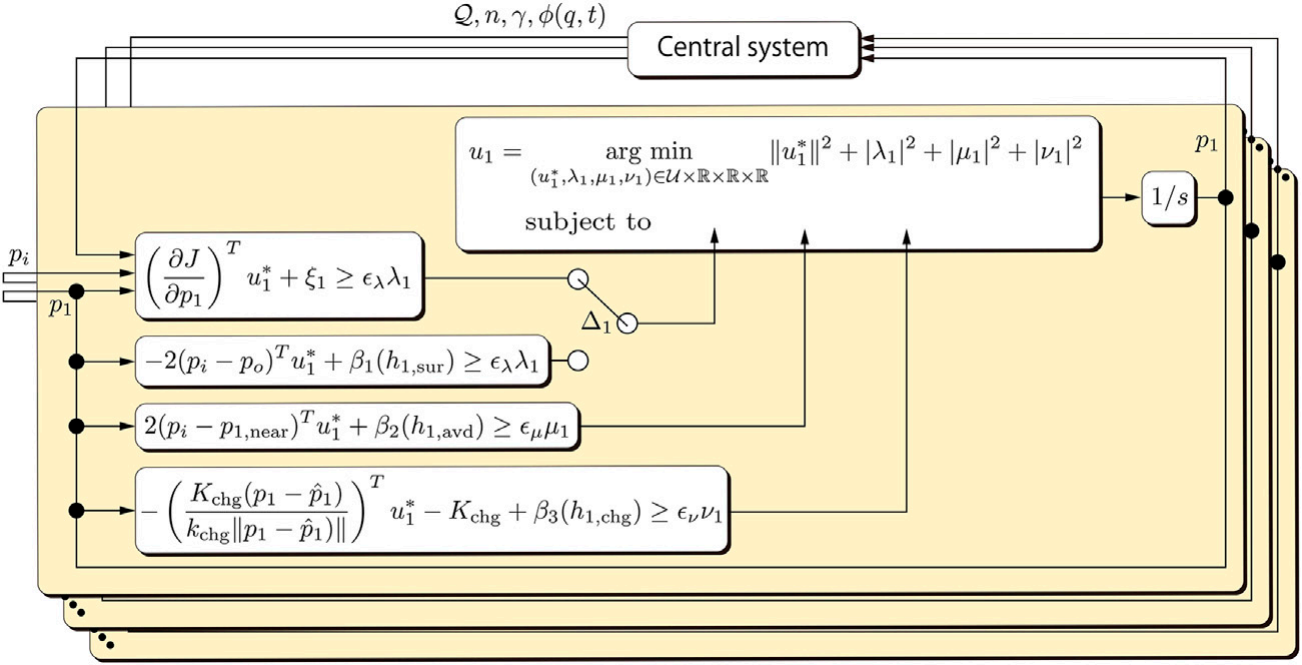
Block diagram of the partially distributed control architecture.

As mentioned before, the sets $S_{i}$ and $E_{i}$ can be locally computed under the network assumed in Section 3. In addition, 17) consists only of local variables/parameters excluding Q , *n*, *γ*, and *ϕ*(*q*, *t*) in $S_{i}$ . In other words, if the field Q , the number of drones *n*, and desired performance level *γ* are shared by the drones, and the density function *ϕ*(*q*, *t*) in $S_{i}$ would be given, each drone *i* can locally solve the optimization problem Eq. 17. It should be now noted that, as assumed in (Hübel et al., 2008; Sugimoto et al., 2015), the density update 6) must be inherently executed by a central system since each drone hardly knows if other drones visited each q∈Q in the past. The overall control architecture is then illustrated in Figure 4. The comprehensive algorithm for drone *i* including landing/takeoff motion and object detection is informally described as Algorithm 1, where *E*_max_ means the battery level at which drones stop charging, and *E*_min_ is the level at which each drone starts landing.

Remark 1 The computation in the density update (6) left to the central computer is almost scalable with respect to the number of drones, while solving the optimization problems (17) for all i at a central computer is not scalable. It is thus fully expected that the present partially distributed architecture works even for large-scale drone networks. Nevertheless, some readers may have a concern about using a central computer itself. In many practical applications, however, the communication infrastructure between drones and a central system is established so that a person at the monitoring center monitors the data acquired by the drones. Thus, assuming the computational supports from the central computer must be reasonable in such application scenarios.

Remark 2 Santos et al. (2019) addressed coverage control with a time varying density function using time-varying CBFs, which is close to the present approach. The contribution of this paper relative to (Santos et al., 2019) is as follows. The controller presented in (Santos et al., 2019) is designed based on the distance between the current robot position and the centroid of the Voronoi cell. However, the relation between the metric and the coverage performance quantified by the objective function is not always obvious. On the other hand, the presented controller The switches between subtasks are also not investigated in (Santos et al., 2019).

Algorithm 1 Algorithm for drone *i*.

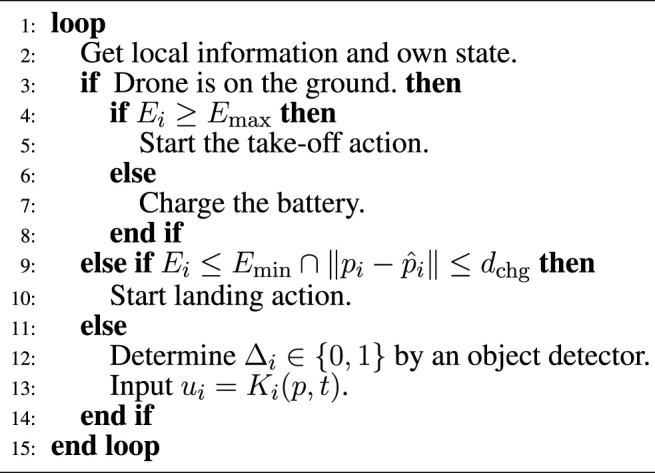

## 5 Simulation

In this section, we focus only on the persistent search mission while ignoring other objectives, object surveillance, safety, and energy persistency. We then verify through simulation that the constrained-based controller achieves the performance specified by the parameter *γ*. To this end, we employ the simplified version of the controller (17):

$$\begin{array}{ll} K_{i}^{\prime }\left(p,t\right) & =\underset{u_{i}^{*}\in R^{2}}{\mathrm{arg min}}\|u_{i}^{*}{\|}^{2} \end{array}$$ (18a)

$$\begin{array}{ll} s.t.\; & {\left(\frac{\partial J\left(p,t\right)}{\partial p_{i}}\right)}^{T}u_{i}^{*}+\xi_{i}\left(p,t\right)\geq 0. \end{array}$$ (18b)

In the simulation, the field is set to Q=[−2,2]m×[−2,2]m . We also let *n* = 3 and the initial positions be selected as *p*_1_ = [−1 0]^*T*^m, *p*_2_ = [1 0]^*T*^m, and *p*_3_ = [1 1]^*T*^m. The altitude *z*_*i*_ and radius *R* of every drone are set to 1.2 and 0.6 m mimicking the experimental testbed that will be presented in the next section. Under the setting, we run the constraint-based controller 18) with *γ* = − 4.0, and compare the performance with the gradient-based controller (Sugimoto et al., 2015), namely *u*_*i*_ = *κ∂J* (*p*, *t*)/*∂p*_*i*_ with *κ* = 5.0 and (Santos et al., 2019). In all of the controllers, we take $\bar{\delta }=0.05$ , $\underline{\delta }=1.0$ and *b* = − 1.0. Remark now that (Santos et al., 2019) does not consider limitation of the sensing radius, but we impose the same limitation as the other two methods by just changing Voronoi cells to *r*-limited ones in order to fairly compare the methods. The gradients of the centroids of the *r*-limited Voronoi cells needed for implementing (Santos et al., 2019) are numerically computed.

**FIGURE 5:**
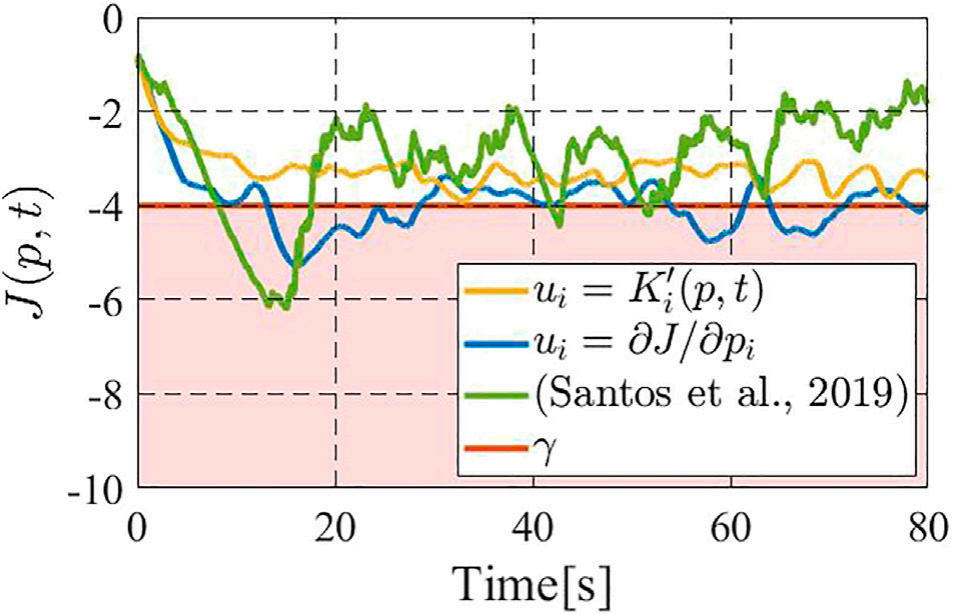
Comparison on the value of the performance function (with three drones): the gradient-based controller (blue) and (Santos et al., 2019) (green) do not meet *J* ≥ *γ*, while the constraint-based controller (yellow) satisfies.

**FIGURE 6:**
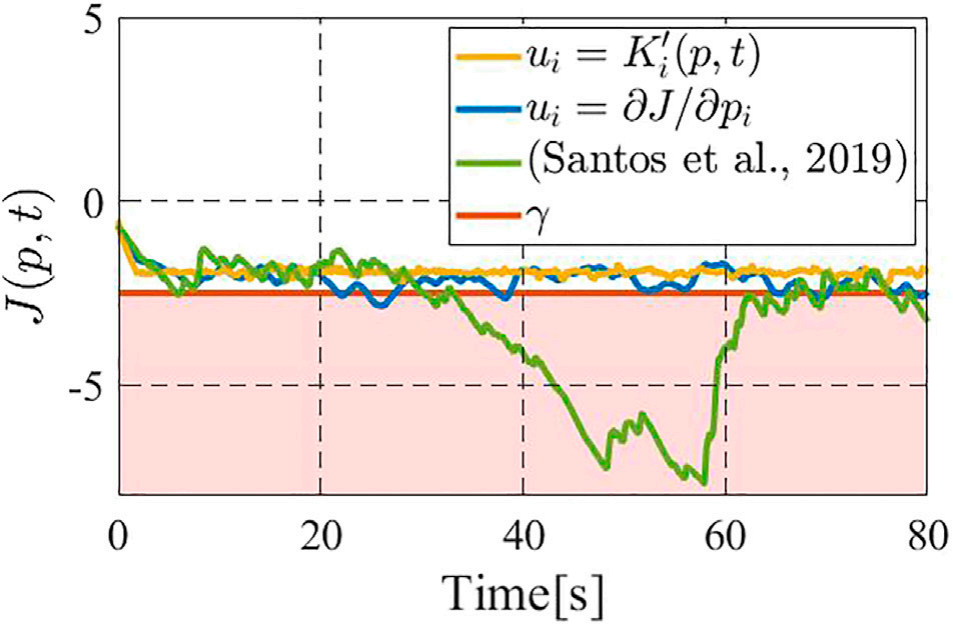
Comparison on the value of the performance function with five drones among the gradient-based controller (blue) (Santos et al., 2019) (green), and the present the constraint-based controller (yellow).

Figure 5 shows the time responses of the performance function *J* for the above two methods, where the blue line shows the performance by the gradient-based controller (Sugimoto et al., 2015), the green line that by (Santos et al., 2019) the yellow line that by the constraint-based controller (18), and the red line illustrates the prescribed performance level *γ* = − 4.0. We see that the gradient-based controller (Sugimoto et al., 2015) and (Santos et al., 2019) occasionally fail to meet the desired performance level, namely the value of performance function *J* goes below *γ*. On the other hand, the constraint-based controller 18) successfully keeps the performance above the level *γ* = − 4.0. Figure 6 illustrates the results for *n* = 5, wherein we take *γ* = − 2.5 to highlight the differences between the present controller and the other two. It is immediate to see that the above insights from Figure 5 are also applied to this case. It is now to be noted that, if we remove the density update 6) from consideration, the controller in (Santos et al., 2019) is itself fully distributed, while the present constraint-based controller still needs partial support from a central computer. However, in the present scene, 6) needs to be executed in a central computed regardless of the control algorithm, as mentioned in Section 4.

**FIGURE 7:**
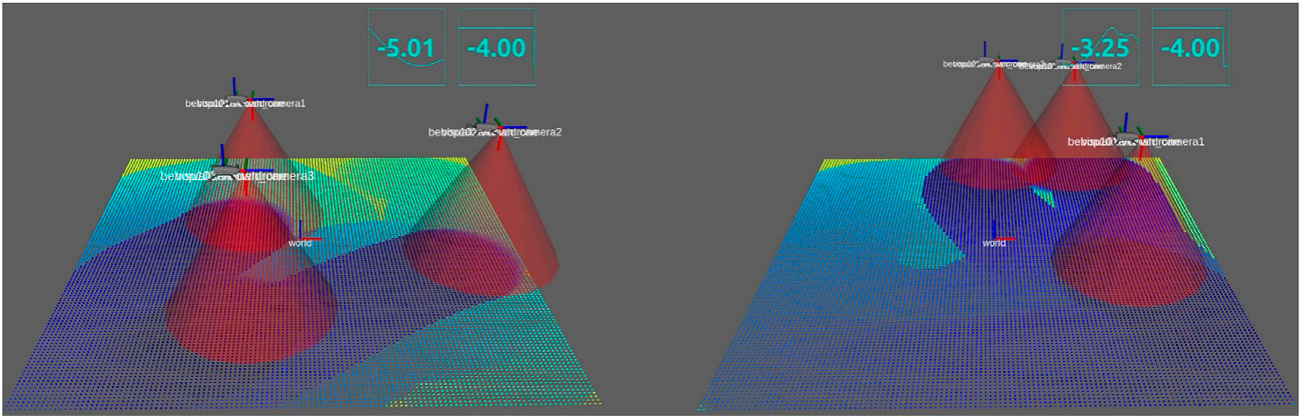
Snapshots at time *t* =17 s: the constraint-based controller **(right)** almost fills the entire field with blue, while some regions remain yellow for the gradient-based controller **(left)**.

Figure 7 shows the snapshot of simulation in Figure 5 at *t* = 17 s, where the left and right figure correspond to the gradient-based controller (Sugimoto et al., 2015) and the constraint-based controller (18), respectively. The color map on the field illustrates the value of the density function *ϕ*(*q*, *t*), where the yellow region has high density while the dark blue means low density. We immediately see from the definition of *J* in Eq. 5 that low density is directly linked with a good search performance. In the left, some areas remain yellow while, in the right, the entire area is almost filled with blue. It is thus concluded that the constraint-based controller 18) achieves a better performance than the gradient-based controller.

Remark that if we take a larger gain *κ*, then the gradient-based controller tends to achieve a better performance and may even meet the prescribed performance level. Even through that, the performance level is not rigorously ensured and, more importantly, it is hard to know an appropriate gain for given environment and parameters in advance. Of course, taking a too large feedback gain may result in unstable motion in real implementation.

It is finally to be noted that the optimization problem in the controller has never been infeasible, namely the gradient $\frac{\partial J\left(p,t\right)}{\partial p_{i}}$ has never been equal to 0 throughout the simulation. Due to pathological cases, the function *h*_*J*_ has not been rigorously proved to be always a time-varying CBF, but it would not matter in practice.

## 6 Experiment

**FIGURE 8:**
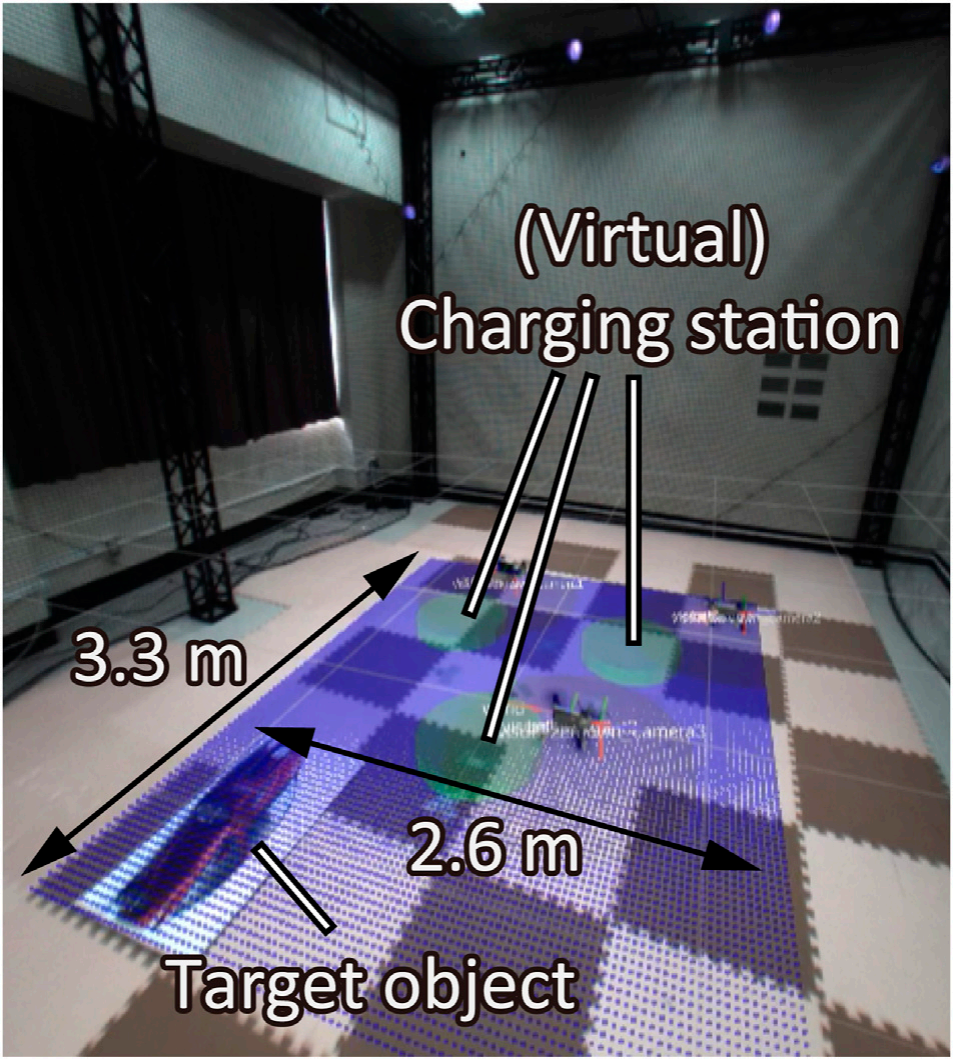
Experiment room: Overview of the environment.

In this section, we demonstrate Algorithm 1 through experiments on a testbed. We set the field Q as a 3.3 m × 2.6 m ground plane as shown in Figure 8. We place a picture of a car on the field as the object to be surveilled. We also employ three Parrot Bebop2 drones (*n* = 3), whose onboard cameras capture the ground plane. We set the virtual charging stations, in which we suppose that drones can charge their batteries.

**FIGURE 9:**
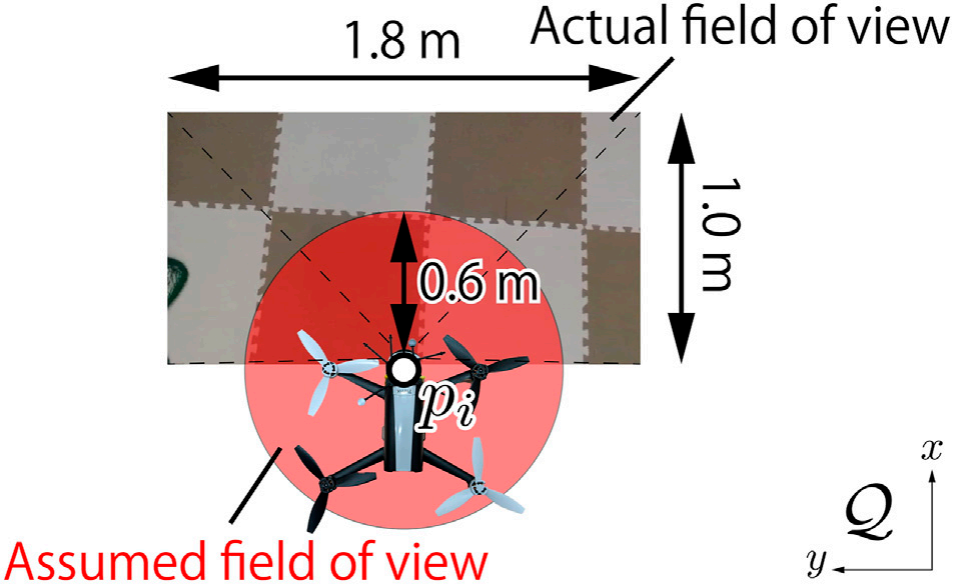
Definition of the field of view for a drone.

A local controller for each drone is designed so that its altitude is maintained to be 1.2 m and the body is parallel to the ground. When a drone takes the above desirable states, the field of view of the camera is given by about 1.8 m × 1.2 m rectangle as illustrated in Figure 9. In order to compensate the gap from the circular field of view assumed in the previous sections, we set the red circle in Figure 9 with radius 0.6 m inside of the rectangle while accepting conservatism. Also, the optical axis of the camera is not perpendicular to the body, which differs from the model in Figure 2. In order to fill the gap, the center of the circle is shifted from that of the rectangle. This shift does not matter in practice since the object position is also shifted in the sequel. Generalization of the algorithm that does not require such remedies is left as a future work.

**FIGURE 10:**
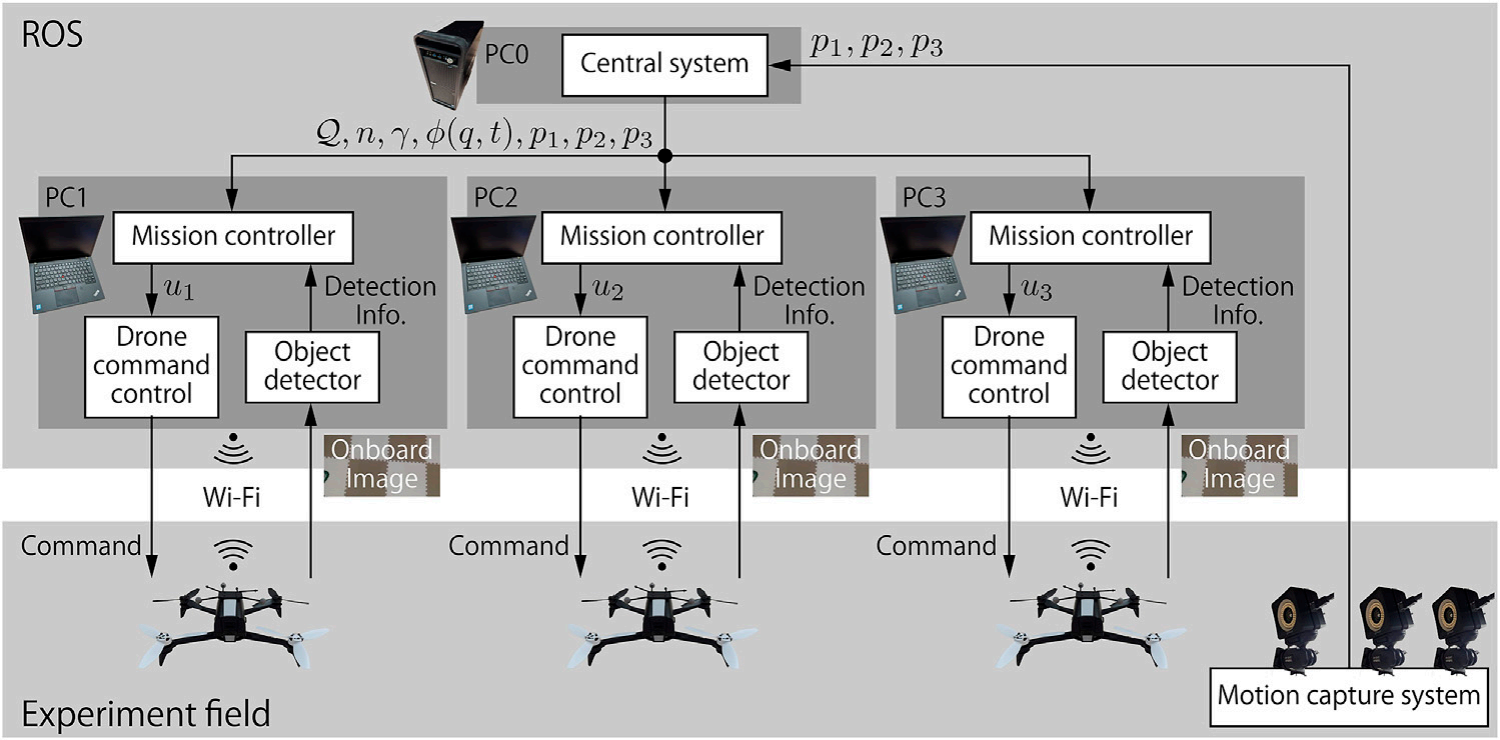
Experiment systems: Schematic description of the system.

The schematic of the testbed is illustrated in Figure 10 which consists of a desktop computer, three laptops, and a motion capture system (OptiTrack) as well as drones. The motion capture measures the positions of drones at every 4.17 ms (240 fps). The desktop computer (PC0) receives all drones’ positions from the motion capture system, updates the value of density function *ϕ*(*q*, *t*), and publishes the positions and the field information such as field size ( Q ), the number of drones (*n*), performance target *γ*, and the current value of *ϕ*(*q*, *t*), to each laptop. Each laptop (PC1–3) implements the distributed controller *K*_*i*_(*p*, *t*), and outputs the velocity command *u*_*i*_ (*i* = 1, 2, 3) to be sent to each drone. The laptops are connected to individual drones by Wi-Fi communication. Each laptop receives the onboard camera images from the drone in real time. It then detects the object by using the tensorflow object detector (https://github.com/osrf/tensorflow_object_detector). The object position is computed by the detection result and the geometric relation, and then shifted to compensate the gap between the rectangle and red circle in Figure 9. The laptop then calculates the inputs *u*_*i*_ based on the information published by PC0 and the detected object position by *Python* script. The quadratic program in Eq. 17 is solved in the script using CVXOPT. The input is converted into a suitable format for communication and sent to the drone. Note that each distributed controller needs the positions of not all drones but only the neighboring drones within the radius 2*R* = 1.2 m. To mimic the real distributed computation, each laptop deletes drones’ positions not within the radius, and does not use the information at all in the program.

The weight of constraints are given as follows:

$$\epsilon_{\lambda }>\epsilon_{\nu }>\epsilon_{\mu }=0.$$

This means that the primary constraint is safety, namely the collision avoidance, and it is treated as a hard constraint. The secondary is the battery charging, and tertiary is the subtasks: the persistent search and object surveillance, which are treated as soft constraints. For the safety reason, we restrict the speed of drones by setting input space U as [ − 0.3 0.3]m*/*s × [ − 0.3 0.3]m/s. The other parameters needed for implementing Algorithm 1 are listed in Table 1.

**TABLE 1:** Parameter setting.

*b*	−1.0	*γ*	−2.0	*E*_min_	1,500	*ϵ*_*λ*_	0.05
*K*	0.3	*d*_sur_	0.1	*k*_chg_	0.15	*ϵ*_*μ*_	0
$\bar{\delta }$	0.05	*d*_avd_	0.5	*d*_chg_	0.3	*ϵ*_*ν*_	0.01
$\underline{\delta }$	1.0	*K*_chg_	25	*E*_max_	4,000

**FIGURE 11:**
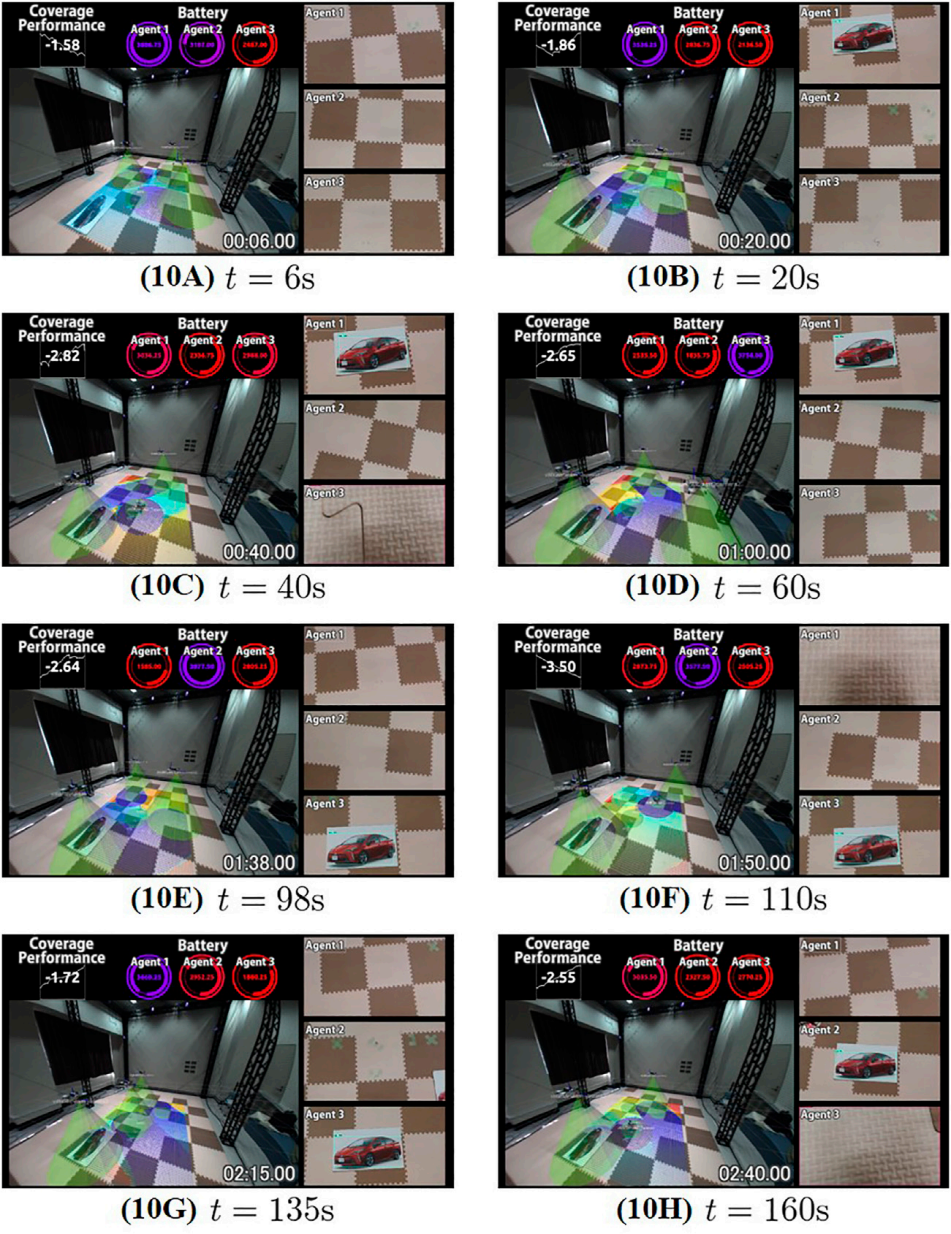
Snapshots of the experiment, where the plane Q (rectangle), charging station (green cylinder) the shifted field of views (green cone) are overlaid. The current value of the performance function *J*, the each state of charge, and the onboard camera views of the drones are also tiled. Note that the shifted field of view and actual camera view do not match perfectly due to the differences shown in Figure 9. In **(C)**, **(F)**, and **(H)**, the drones land on the ground for charging. The object (picture of car) is monitored by one of the drones in all scene except **(A)**.

The snapshots of the experiment are shown in Figure 11. When the object is not detected and all drones’ batteries have enough states of charge, drones run the persistent search and move around over the plane Q (Figure 11A). In Figure 11B, drone 1 successfully detects the object. Accordingly, it switches to the subtask of the object surveillance, and stays above the object. Meanwhile, other drones continue the persistent search. We also see from Figure 11C that, when the battery level of drone 3 is low, it returns to and lands on the charging station. After charging, he restarts the subtasks (Figure 11D). Through the experiment, every drone autonomously repeats these actions depending on the situation (Figures 11E–H). It is to be emphasized that the drones never crash against each other through the experiment owing to the primary constraint.

**FIGURE 12:**
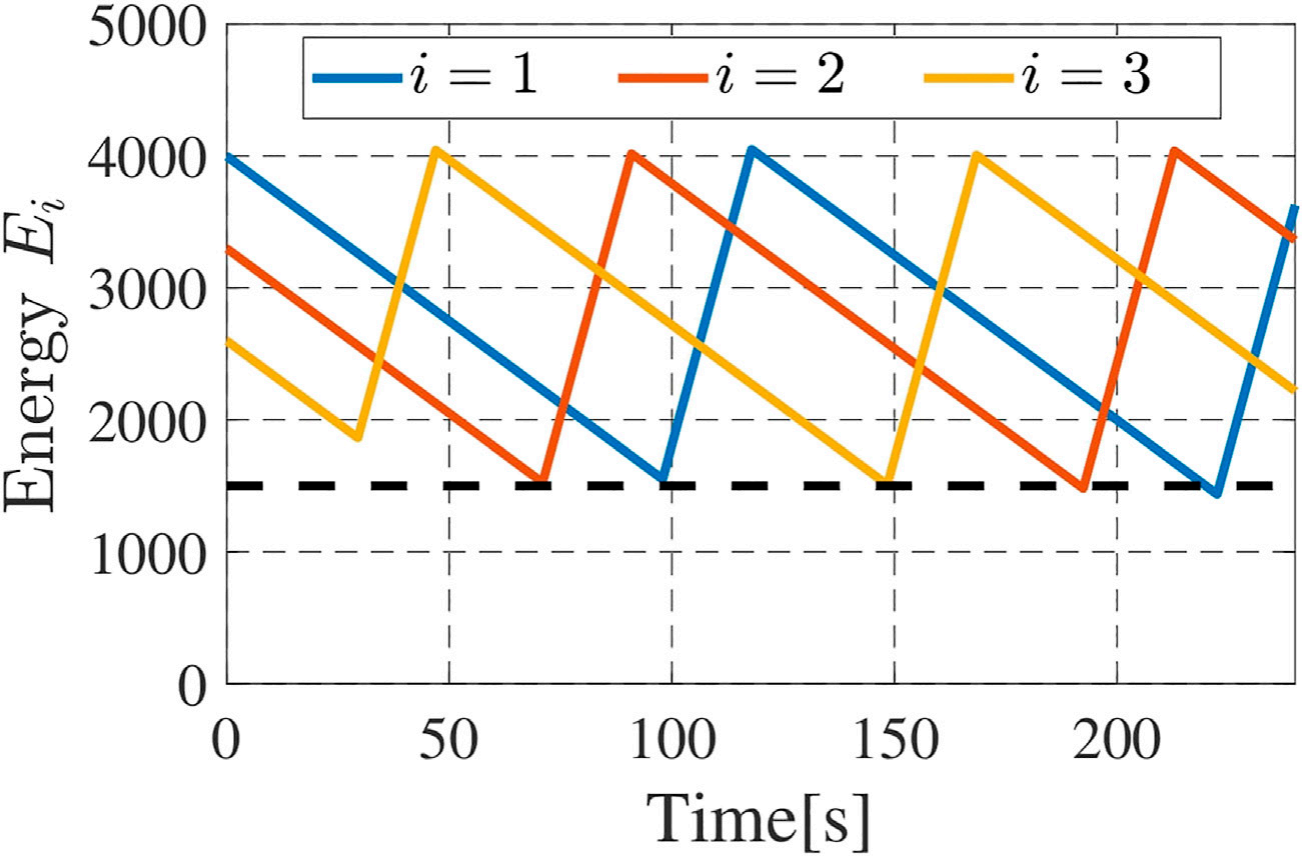
Time series of *E*_*i*_. Each drone recharges its battery before *E*_*i*_ reaches the minimum limit.

**FIGURE 13:**
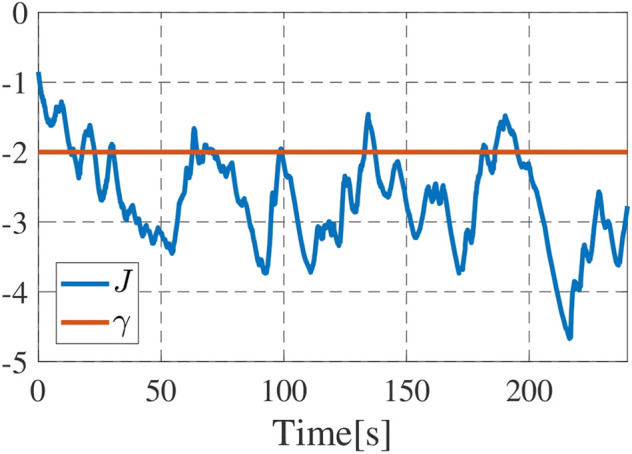
Time series of *J*, where the blue line denotes the function *J* and red line does its target level *γ*.

Let us next confirm the function of the secondary constraint for the energy persistency. The time series of the (virtual) states of charge are shown in Figure 12. We see from the figure that the drones successfully return to the charging station, and recharge the battery before their batteries reach the minimum limit *E*_min_ shown by dashed line with slight exception at around *t* = 225s. Finally, Figure 13 shows the time series data of the value of the function *J*. We see that the drones frequently failed to satisfy the performance level *γ*. This is fully reasonable since the collision avoidance, energy persistency and object surveillance are prioritized over the subtasks of persistent search. We see that the performance level is high in the early stage, where all drones engage in the persistent search as seen in Figure 11A. The performance decreases at around *t* = 20s since a drone switches to object surveillance (Figure 11B). The performance further decays around *t* = 30–40s since a drone goes back to the charging station and only one drone engages in persistent search (Figure 11C). Once the drone restarts persistent search (Figure 11D), the performance improves during *t* = 60–80 but it again decays at around *t* = 80s since another drone returns to the station. It is thus concluded that the present prioritization works as expected, and the present algorithm autonomously completes the overall mission.

## 7 Conclusion

In this paper, we have investigated a persistent object search and surveillance mission with safety certificates for drone networks. To address the issue, the control goals for the persistent object search and surveillance together with certificates for safety and energy persistency have been rigorously formulated in the form of constraint functions. To design a controller that fulfills the constraints, we have derived inequality constraints to be met by the control input, following the manner of CBFs. We then have presented a constraint-based controller that appropriately prioritizes constraints to manage conflicts among specifications. The simulation study has revealed that the constraint-based controller certifies a prescribed performance level for the searching mission, differently from the authors’ antecessor and other related publications. The present algorithm has also been demonstrated through experiments. In the experiment, it has been confirmed that safety and energy persistency are successfully guaranteed by the controller even in the presence of a variety of uncertain factors in the real physical world, not in the ideal mathematical models. We have also observed through experiments that the present prioritization of the specifications works as expected, namely drones prioritize safety and energy persistency at the cost of the control goals for persistent object search and surveillance.

## Data Availability

The original contributions presented in the study are included in the article/Supplementary Material, further inquiries can be directed to the corresponding author.

## Supplementary Material

Supplementary Material Experimental movie of the present constraint-based controller.
